# Supplementary material for: Quaternization-spiro design of chlorine-resistant and high-permeance lithium separation membranes
Source: Nat Commun. 2023 Sep 7;14:5483. doi: 10.1038/s41467-023-41169-x (PMC10482931; doi:10.1038/s41467-023-41169-x)
Supplement: Supplementary file 1 — Supplementary Information [file 41467_2023_41169_MOESM1_ESM.pdf]

Supplementary Information

Quaternization-spiro design of chlorine-resistant and high permeance lithium separation membranes

Huawen Peng[1], Kaicheng Yu[2], Xufei Liu[1], Jiapeng Li[1], Xiangguo Hu[2], Qiang Zhao[1]*

[1]Key Laboratory of Material Chemistry for Energy Conversion and Storage, (Ministry of Education), School of Chemistry and Chemical Engineering, Huazhong University of Science and Technology, Wuhan 430074, P. R. China. E-mail: zhaoq@hust.edu.cn

[2]National Engineering Research Center for Carbohydrate Synthesis, Jiangxi Normal University, Nanchang 330022, P. R. China.

1. Supplementary Methods

1.1 Simulations of molecule diffusion (Supplementary Fig. 5). All the all-atom molecule dynamic simulations were based on a general amber force field with the restrained electrostatic potential charges[1]. The system is a relaxed liquid configuration at 298 K. The total run time was 10 ns NPT for the equilibrium simulation. We used the relaxed system as a starting configuration. As it is prior to system relaxation molecule dynamic, energy minimization was carried out with a composite protocol of steepest descent using termination gradients of 100 kJ mol^{-1} nm^{-1}. The Nose ́-Hoover thermostat[2] was used to maintain the equilibrium temperature at 298 K and periodic boundary conditions were imposed on all three dimensions. The Particle Mesh-Ewald method[3] was used to compute long-range electrostatics within a relative tolerance of 1×10^{-6}. A cut-off distance of 1 nm was applied to real-space Ewald interactions. The same value was used for van der Waals interactions. The linear constraint solver algorithm[4] was applied to constrain bond lengths of hydrogen atoms. A leap-frog algorithm[5] was used with a time step of 2 fs.

1.2 Simulations of free volume (Fig. 2d, e). Initially we loaded periodic cubic cells with dimensions of 6 nm. Polymerization steps were then performed between reactive groups (-NH and -COCl). To allow the structure to adapt a general Amber force field with the restrained electrostatic potential charges[1], we controlled a cutoff of 0.6 nm with intermediate 1ns NVT for the molecular dynamics steps[6, 7]. After we got an amorphous structure, the total run time was 10 ns for the equilibrium molecule dynamic simulation. Polymer fractional free volume (FFV) was estimated by the following formula:

$$FFV = \frac{V_f}{V_f + V_0} \quad (1)$$

Where V_f and V_0 are the free volume and occupied volume. The values of V_f and V_0 are explored using probe with radius of 1 Å, which can also give the morphology of voids in aggregate. The cavity size distributions of QSPIP-TMC and PIP-TMC were calculated by Zeo++ software tool[8].

1.3 Simulations of water diffusion behaviors (Fig. 4). The initial structure of polyamide was established at the center of the c-axis of a space box of 105×105×210 Å using area heuristics in the packmol program. 200 TMC and 300 QSPIP (or PIP) were put into simulation box and moved randomly. In the process of NVT dynamics, the judgment of forming amide bonds between TMC and QSPIP is based on the overlapping node of the wave function. That is, when the van der Waals surfaces of amino nitrogen atoms (QSPIP) and acyl carbon atoms (TMC) overlap, then amide bond forms. First-principles molecule dynamic methods with Gromacs[9] was used to study the collision, adsorption and molecular transfer processes of intermolecular interaction. Molecule dynamic algorithm combined with NVT ensemble, Nose-Hover thermostat[10], 0.1 fs step size, 500,000 steps. In the calculation steps, the energy minimization is used to balance the system, and then the Maxwell distribution method is used to give the molecules different collision probabilities.

1.4 Molecular weight cut-off and pore size distribution (Fig. 2c). Polyethylene glycol (PEG) aqueous solution (1000 ppm, Mw: 200, 400, 600, 1000, 1500 Da) were used as feed solution. The PEG concentrations in feed and permeate were determined by the total organic carbon (TOC-L CPN, Shimadzu, Japan). The curve of PEG rejection-Mw was fitted by a log-normal probability function. Molecular weight cut-off of membrane was determined as the molecule weight of PEG when PEG rejection is 90%. The pore size distribution of membranes was determined by the probability density function[11].

1.5 Chlorine-resistance test (Fig. 5a, b). Membranes were immersed in sodium hypochlorite aqueous solution (NaClO, 200 ppm, pH=6) under continuous stirring. The NaClO solution was replaced every 24 h to maintain constant NaClO concentration (200 ppm). After that, the membranes were picked out and washed thoroughly with DI water before test. The chlorine-resistant ability of membranes was evaluated by $MgCl_2$ nanofiltration (1000 ppm, 6 bar).

1.6 Preparation of large-area QSPIP-TMC membrane (Fig. 7a). A piece of PSf membrane (1.0 × 2.0 m^2) was glued on the polyvinyl chloride board and immersed into QSPIP aqueous solution (0.5 wt%, pH=11) for 3 min. The membrane was dried at room temperature, the liquid on the membrane was wiped with filter paper. Then the membrane was immersed into TMC hexane solution (0.1 wt%) for 1 min. After that, the membrane was dried at room temperature for 24 h.

1.7 Preparation of QSPIP-TMC module (Fig. 7c). First, the large-area QSPIP-TMC membrane from Fig. 7a was cut into pieces of 80 × 27 cm^2. Then three pieces of membrane were sandwiched with the feed spacer (27.5 × 40.0 cm^2, thickness: 1.7 cm) and permeate spacer (27.5 × 46.0 cm^2, thickness: 1.0 cm) and rolled into a spiral wound module (Effective area: ~0.5 m^2). The schematic of spiral wound modules was shown in Fig. 7c (top). The module was sealed into a membrane shell for nanofiltration test.

2. Supplementary figures

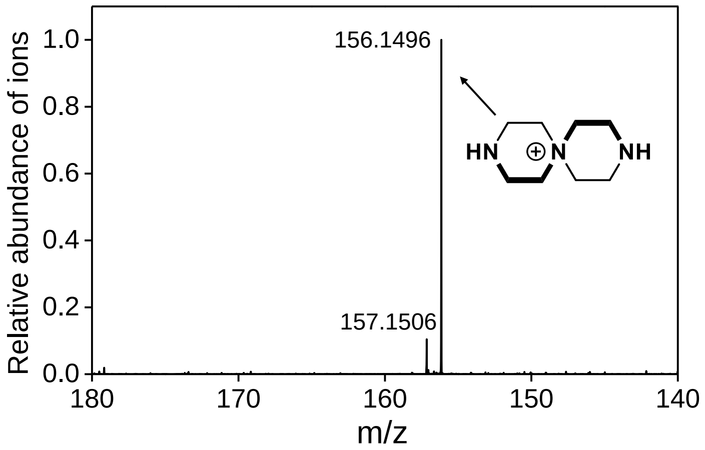

Supplementary Fig. 1. The mass spectroscopy of QSPIP.

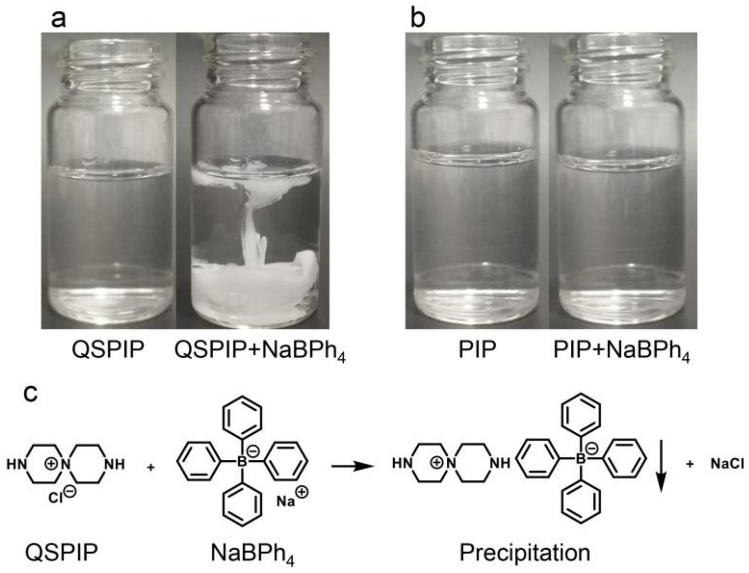

Supplementary Fig. 2. Determination of positive charge of QSPIP. (**a**) Photographs of QSPIP solution (0.5 wt%) and that added with sodium tetraphenylborate ($NaBPh_4$, 0.5 wt%) aqueous solution; (**b**) photographs of PIP aqueous solution (0.5 wt%) and that added with $NaBPh_4$ aqueous solution (0.5 wt%). (**c**) Counter ion exchange between QSPIP and $NaBPh_4$.

Discussions. When $NaBPh_4$ solution was dropped in QSPIP solution, the white precipitation appears immediately. This is because the chloride anions of QSPIP were replaced by BPh_4^- anions, which are more hydrophobic compared to chloride anions. By contrast, no precipitation appears when $NaBPh_4$ was dropped in PIP solution.

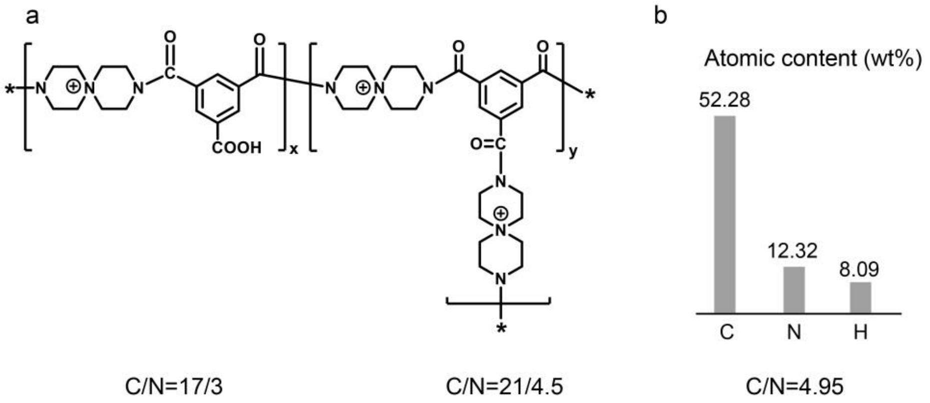

Supplementary Fig. 3. The crosslinking degree of QSPIP-TMC freestanding film. (**a**) Chemical structure of QSPIP-TMC. (**b**) The elemental content of QSPIP-TMC freestanding film characterized by organic elemental analyzer. Please note: the crosslinking degree of polyamide network was defined as the proportion of cross-linked structure (y), in which all acyl chlorides of TMC react with amine group of QSPIP.

The crosslinking degree (y) of QSPIP-TMC is calculated as:

$$\frac{17}{3}x + \frac{21}{4.5}y = 4.95 \tag{2}$$

$$x + y = 1 \tag{3}$$

The y is 72%, which is close to the crosslinking degree of PIP-TMC TFCM in literatures[12, 13].

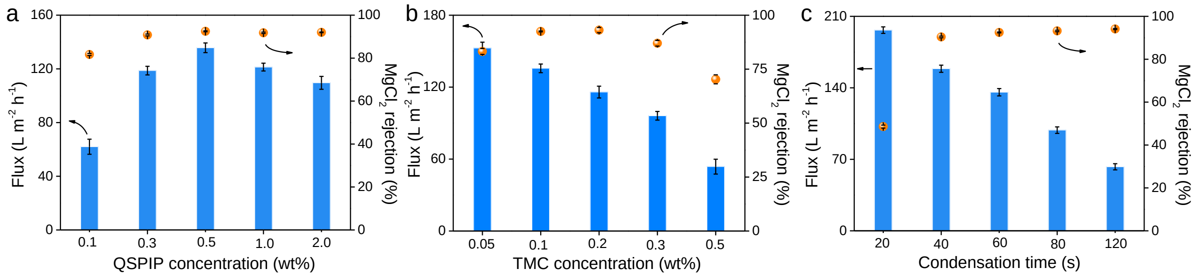

Supplementary Fig. 4. Effect of membranes preparation conditions in separation performance. (**a**) Effect of QSPIP concentration in separation performance of QSPIP-TMC TFCM. (TMC: 0.1 wt%, condensation time: 60 s). (**b**) Effect of TMC concentration in separation performance of QSPIP-TMC TFCM. (QSPIP: 0.5 wt%, condensation time: 60 s). (**c**) Effect of condensation time of QSPIP/TMC in separation performance of QSPIP-TMC TFCM. (QSPIP: 0.5 wt%, TMC: 0.1 wt%). Test conditions: 1000 ppm $MgCl_2$, 6 bar, 30 °C.

Discussions. On basis of the supplementary Fig. 4, concentration of QSPIP and TMC were kept at 0.5 wt% and 0.1 wt%, and the condensation time of QSPIP/TMC was 60 s.

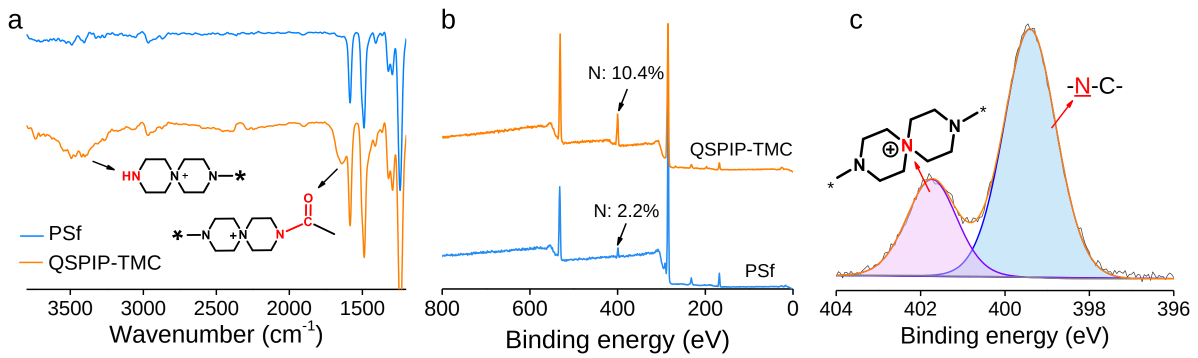

Supplementary Fig. 5. Characterizations of chemical structures of membranes. (**a**) The ATR-FTIR, (**b**) XPS characterizations of PSf and QSPIP-TMC membrane. (**c**) The N 1s core level spectra of QSPIP-TMC membrane.

Discussions. The QSPIP-TMC composite membrane shows signals of both the amine (3460 cm^{-1}, from QSPIP) and amide group (1640 cm^{-1}, due to the QSPIP-TMC condensation) in ATR-FTIR spectra. The N atomic content of QSPIP-TMC membrane is 10.4%, 4.7 times higher than PSf membrane. The XPS peak at 401.7 eV corresponding to quaternary ammonium group is seen on QSPIP-TMC membrane. These results are consistent with the QSPIP-TMC freestanding membrane (Fig. 1, main text).

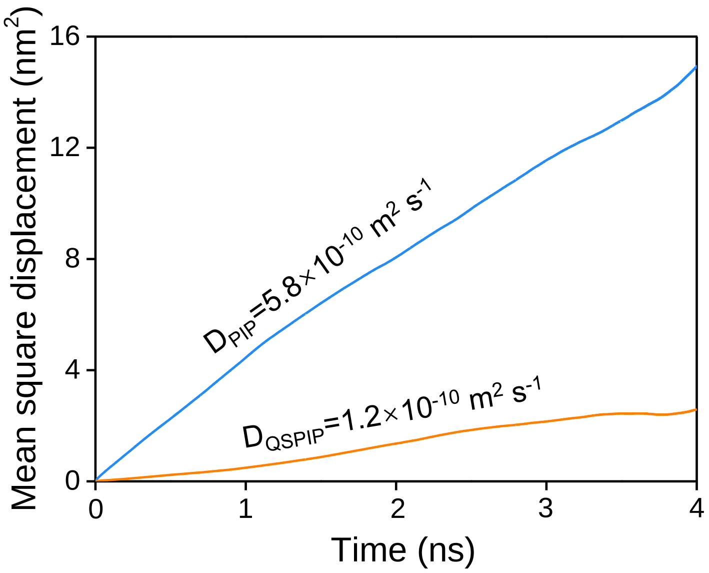

Supplementary Fig. 6. The mean square displacement of QSPIP and PIP in hexane with time.

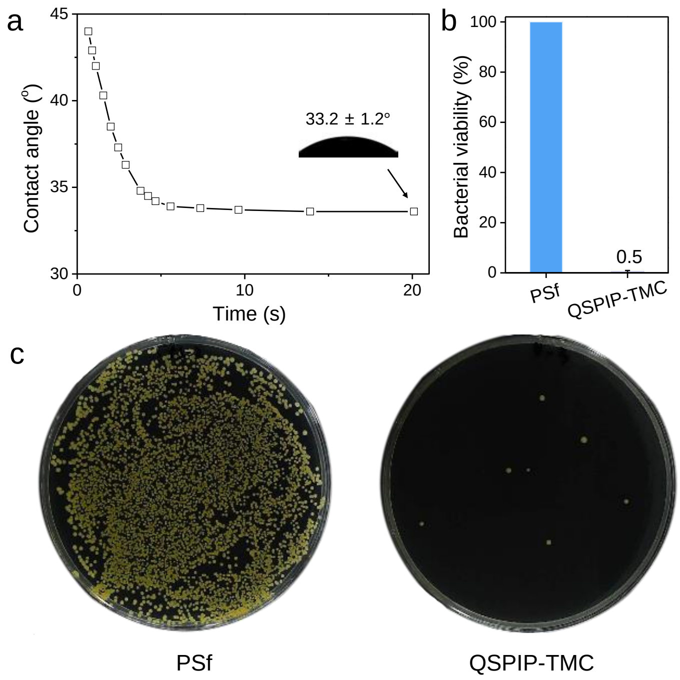

Supplementary Fig. 7. Characterizations of antibacterial property of QSPIP-TMC. (**a**) The dynamic water contact angle of QSPIP-TMC membrane. (**b**, **c**) The antibacterial property of PSf and QSPIP-TMC membrane towards *S. aureus*.

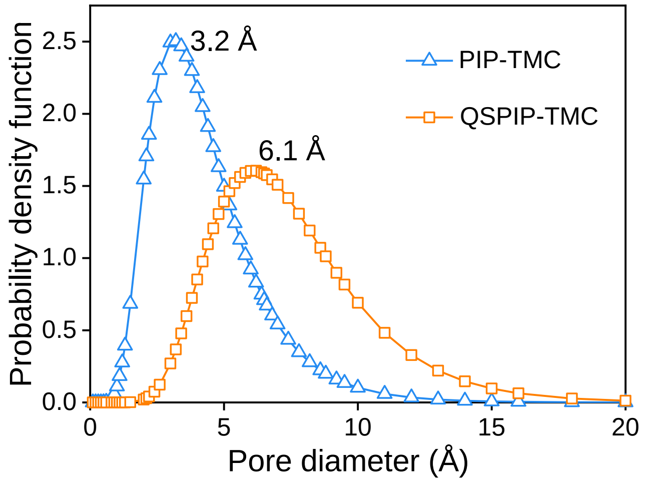

Supplementary Fig. 8. The mean pore diameter of PIP-TMC and QSPIP-TMC membranes obtained from PEG rejection (Fig. 2c).

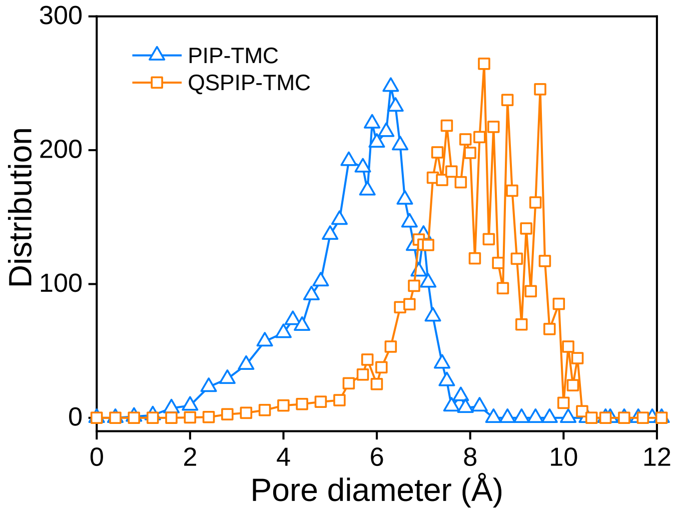

Supplementary Fig. 9. The pore diameter of QSPIP-TMC and PIP-TMC network obtained from molecule dynamic simulations (Fig. 2d, e).

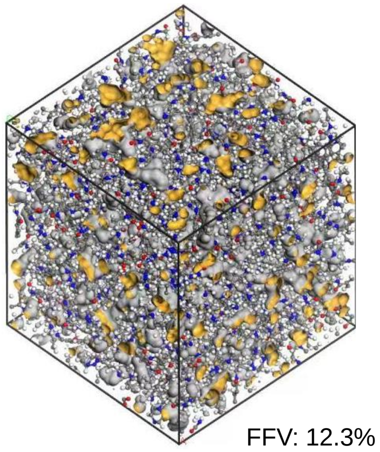

Supplementary Fig. 10. Molecular dynamics simulation cell of free volume of BAPP-TMC. The fractional free volume of BAPP-TMC is 12.3%, which is half that of QSPIP-TMC (24.0%). This is due to the spiral configuration of QSPIP that impedes the inefficient packing of polymer chain. The BAPP-TMC membrane shows lower permeance (2.4 L m^{-2} h^{-1} bar^{-1}) than QSPIP-TMC (23.0 L m^{-2} h^{-1} bar^{-1}).

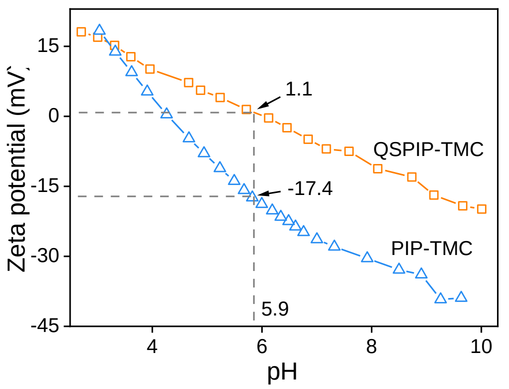

Supplementary Fig. 11. Zeta potential of QSPIP-TMC and PIP-TMC membranes.

Please note: pH of the feed (1000 ppm $MgCl_2$) is 5.9. At this pH, the zeta potential of QSPIP-TMC and PIP-TMC TFCMs are 1.1 and -17.4 mV, respectively. The positive charge of QSPIP-TMC membrane is enhanced due to the quaternary ammonium of QSPIP.

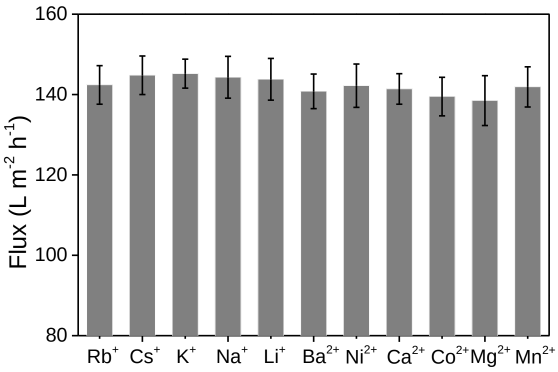

Supplementary Fig. 12. Flux of QSPIP-TMC TFCM at 6 bar. (feed salts: 1000 ppm)

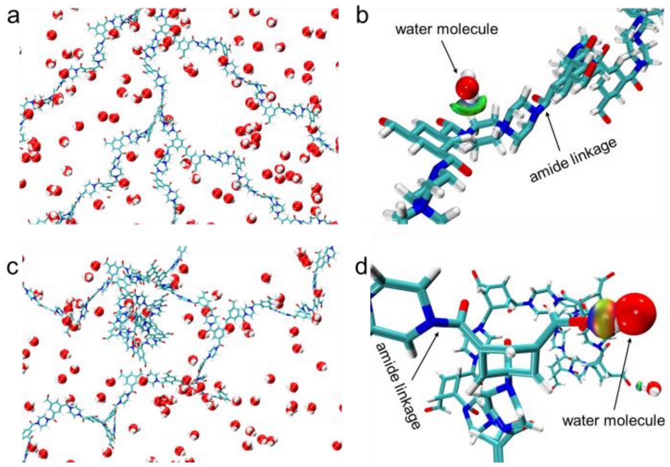

Supplementary Fig. 13. The model structure (segment) of (**a**, **b**) QSPIP-TMC, (**c**, **d**) PIP-TMC in molecular simulation (Fig. 4a). Water molecules diffuse through the polyamide networks and their diffusion behaviors were collected.

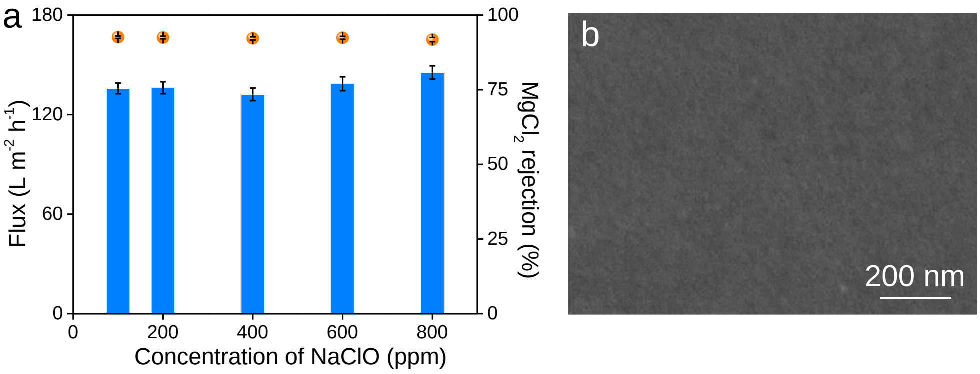

Supplementary Fig. 14. Chlorine resistance of QSPIP-TMC. (**a**) Effect of NaClO concentration in the separation performance of QSPIP-TMC membrane. (Immersion time: 48 h, test conditions: 1000 ppm $MgCl_2$, 6 bar). (**b**) The surface morphology of QSPIP-TMC membrane after being treated by 800 ppm NaClO for 48 h.

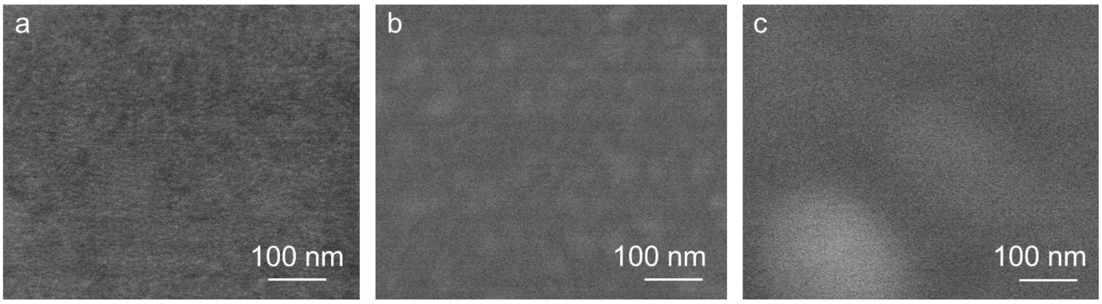

Supplementary Fig. 15. The surface morphologies of pristine membranes: (**a**) QSPIP-TMC, (**b**) PEI-TMC and (**c**) BAPP-TMC membrane (without NaClO treatment).

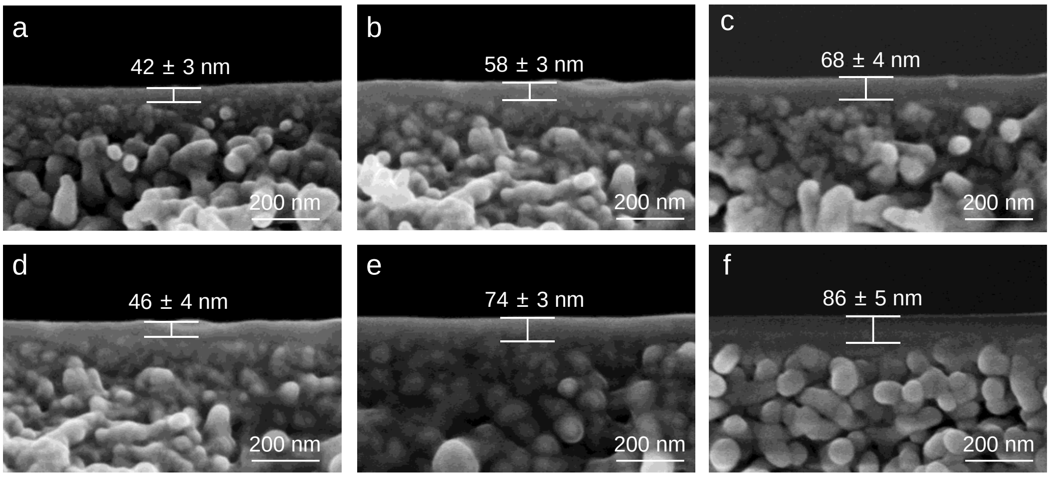

Supplementary Fig. 16. Thickness of membranes. Thickness of (**a**,**d**) QSPIP-TMC, (**b**,**e**) PEI-TMC, (**c**,**f**) BAPP-TMC TFCMs before (**a**-**c**) and after (**d**-**f**) chlorine treatment for 400 h.

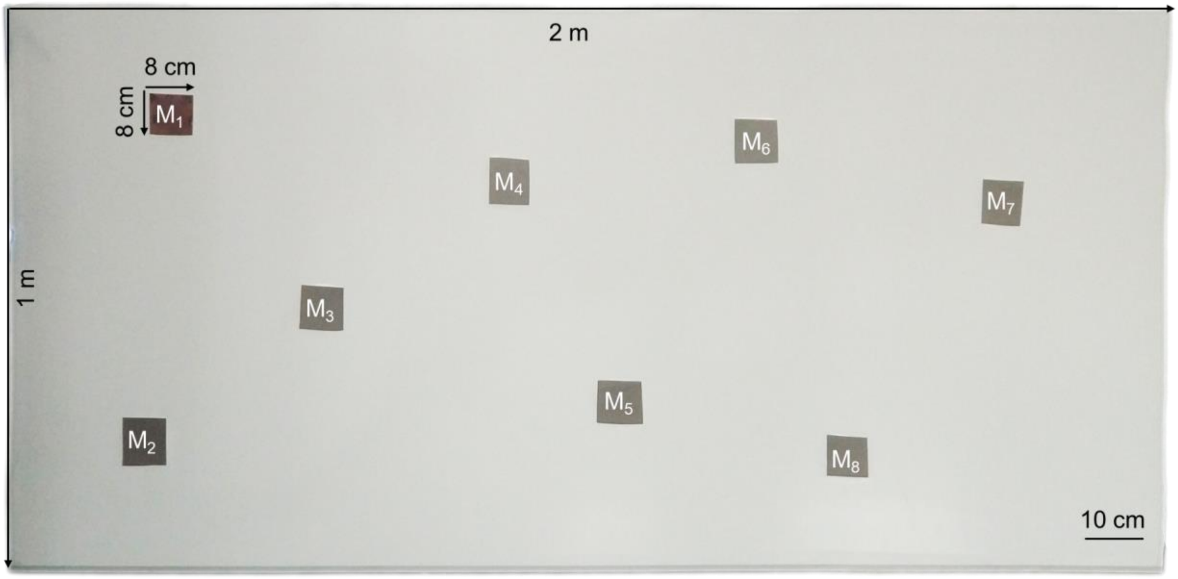

Supplementary Fig. 17. Optical image of membranes (M_1-M_8, 8 × 8 cm^2) selected from large-area QSPIP-TMC membrane (1 × 2 m^2) for nanofiltration test or antichlorine test. Please note: a new large QSPIP-TMC membrane (1 × 2 m^2) was used to prepare modules.

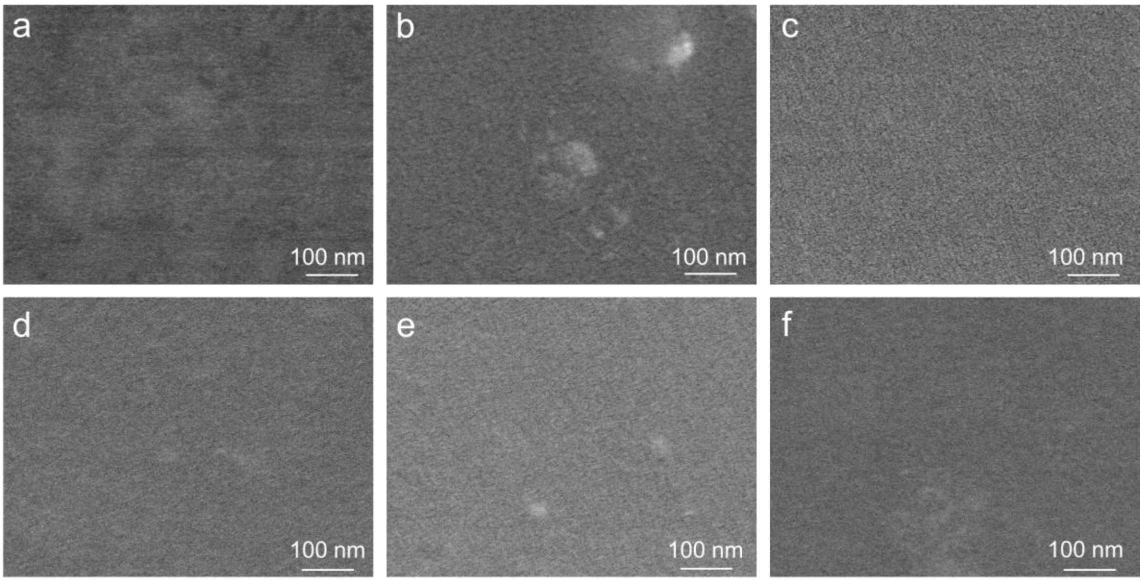

Supplementary Fig. 18. Surface morphologies of (**a**) M_0 and (**b-f**) M_1-M_5 membrane. Note: M_0 was the small flat-sheet membrane (*i.e.*, that in Fig. 3 in the main text). M_1-M_5 were randomly selected from the large-area QSPIP-TMC membrane in Fig. 7a, which was also shown in Supplementary Fig. 17.

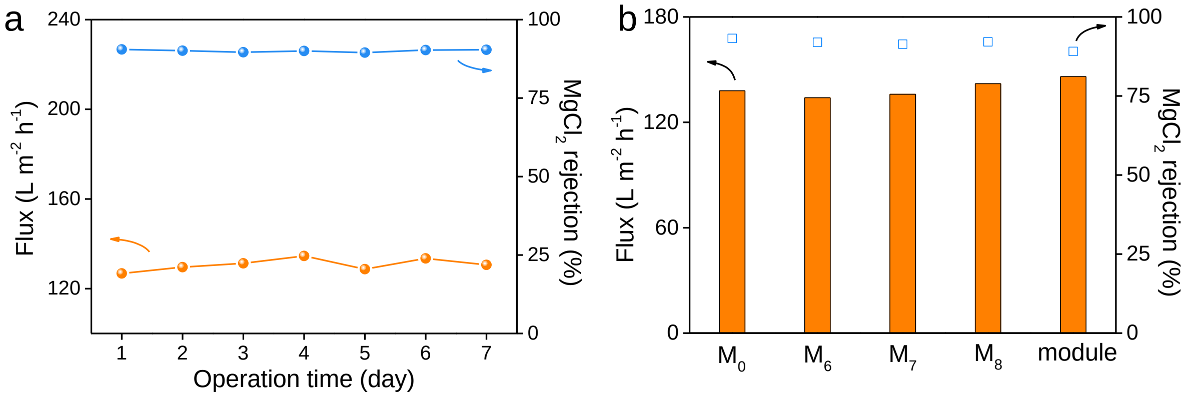

Supplementary Fig. 19. Separation performance of the large-area membranes and modules. (a) Separation performance of QSPIP-TMC module during 7-day nanofiltration test. (**b**) Separation performance of large-area QSPIP-TMC membrane and modules before and after being treated by 200 ppm NaClO for 48 h. Note: M_6-M_8 was randomly selected from the large-area QSPIP-TMC membrane in Fig. 7a. (Test conditions: 1000 ppm $MgCl_2$, 6 bar).

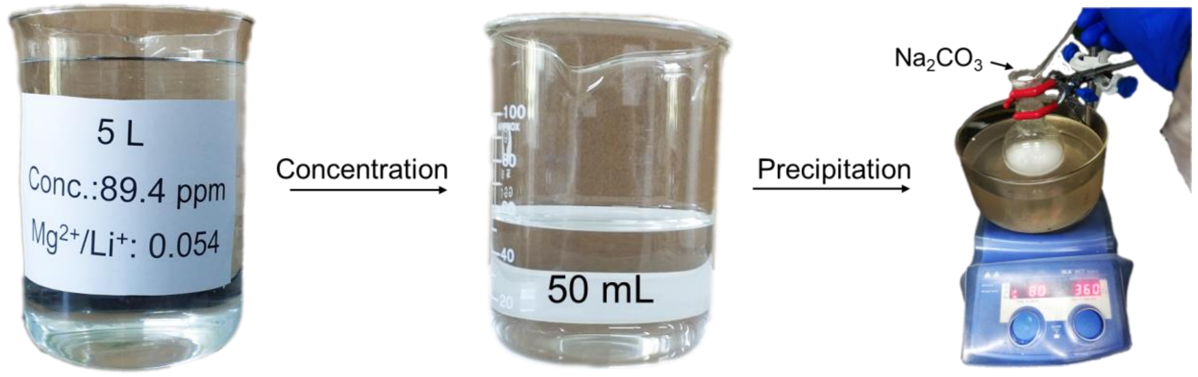

Supplementary Fig. 20. Preparing Li_2CO_3 product from the permeate of *3rd*-stage nanofiltration. The permeate was concentrated until the mass content of Li_2CO_3 was ~2 wt%. Then the excessive Na_2CO_3 was added into solution to precipitate Li_2CO_3 at 80 °C. The product was filtered, washed with 80 °C DI water and dried in vacuum oven (50 °C, 12 h).

3. Supplementary tables

Supplementary Table 1. Original data for performance comparison in Fig. 3g

Membrane	C_{feed} (ppm)	Mg^{2+}/Li^{+} ratio in feed	Permeance (LMH/bar)	Mg^{2+}/Li^{+} selectivity	Ref
PIP-MWCNTs	2000	21.4	14	7.1	14
PEI-TMC	2000	20	5.2	20	15
BPEI/TMC/EDTA	2500	24	0.6	9.2	16
PEI/TMC/CNC-COOH	2000	30	4.2	12.2	17
PEI/TMC/CNC-COOH	2000	60	3.4	5.8	17
DAPP-TMC	2000	20	2.5	2.6	18
PHF-doped TFC	2000	21.4	6.3	13.1	19
[MimAP][Tf_2N]-PA	2000	20	4.7	8.1	20
(PES-GO)/PEI/TMC	2000	20	11.2	16.1	21
Dual-skin layer NF	2000	21.4	12	33.4	22
MBCN-0.02	2000	73	5.6	23.9	23
PEI/GQDs-NH2/ TMC	2000	20	11.9	27.8	24
PES/CQDs-NH2TMC	2000	20	12	14.4	25
Cu−MPD membrane	2000	23	16.3	8	26
PEI-TMC-QBPD	2000	50	13.6	5.9	27
PEI-TMC-HMTAB	2000	50	16.3	10.2	28
PEI@15C5	2000	20	8	14	29
PIL-TMC	2000	100	10	9.8	30
PBI_12-25K	2000	10	2.8	15.2	31
PEI-LDH/GA/PAN	1000	10	4	18.7	32
IP membrane	2000	20	0.4	3.3	33
SERS-0.50	2000	20	2.3	7.7	33
SIP-0.15	2000	20	1.3	15.4	33
(MWCNTs-COOK)-PEI	2000	20	12.2	58	34

PEI-TMC-QEDTP	2000	50	18.8	15.6	35
QSPIP-TMC	2000	100	22.2	9.1	This work

4. Computational Details (Fig. 6b-d)

Calculations were performed using the Gaussian 09 software package. All the geometries and energetics were fully optimized at the B3LYP/6-311+G (d, p)[36, 37] level of theory. Single-point energy calculations on the optimized geometries were evaluated using Truhlar and co-workers' B3LYP and 6-311+G (d, p) basis set within the solvation model density (water)[38]. The thermal corrections evaluated from the unscaled vibrational frequencies at the B3LYP /6-311+G (d, p) level on the optimized geometries were then added to the B3LYP /6-311+G (d, p) electronic energies to obtain the free energies. The structural representations were generated with Gaussian View. Note: the source data are provided in the submitted excel file.

Supplementary References

1. Wang, J. M., Wolf R. M., Caldwell J. W., Kollman P. A. & Case D. A. Development and testing of a general amber force field. *J. Comput. Chem.* **25**, 1157 (2004).
2. Berendsen, H. J. C., Postma J. P. M., van Gunsteren W. F., DiNola A. & Haak J. R. Molecular dynamics with coupling to an external bath. *J. Chem. Phys.* **81**, 3684-3690 (1984).
3. Astrakas, L. G., Gousias C. & Tzaphlidou M. Structural destabilization of chignolin under the influence of oscillating electric fields. *J. Appl. Phys.* **111**, 074702 (2012).
4. Hess, B., Bekker H., Berendsen H. J. C. & Fraaije J. LINCS: A linear constraint solver for molecular simulations. *J. Comput. Chem.* **18**, 1463 (1997).
5. Van Gunsteren, W. F. & Berendsen H. J. C. A Leap-frog Algorithm for Stochastic Dynamics. *Molec. Simulation* **1**, 173-185 (2007).
6. Hess, B., Kutzner C., van der Spoel D. & Lindahl E. GROMACS 4: Algorithms for highly efficient, load-balanced, and scalable molecular simulation. *J. Chem. Theory Comput.* **4**, 435 (2008).
7. Abbott, L. J., Hart K. E. & Colina C. M. Polymatic: a generalized simulated polymerization algorithm for amorphous polymers. *Theor. Chem. Acc.* **132**, 1334 (2013).
8. Willems, T. F., Rycroft C., Kazi M., Meza J. C. & Haranczyk M. Algorithms and tools for high-throughput geometry-based analysis of crystalline porous materials. *Microporous and Mesoporous Mater.* **149**, 134-141 (2012).
9. Ataka, K.-i., Yotsuyanagi T. & Osawa M. Potential-dependent eeorientation of water molecules at an electrode/electrolyte interface studied by surface-enhanced infrared absorption spectroscopy. *J. Phys. Chem.* **1996**, 10664-10672 (1996).
10. Hoover, W. G. & Holian B. L. Kinetic moments method for the canonical ensemble

distribution. *Phys. Lett. A* **211**, 253-257 (1996).

11. Yuan, B. B., Zhao S. C., Hu P., Cui J. B. & Niu Q. J. Asymmetric polyamide nanofilms with highly ordered nanovoids for water purification. *Nat. Commun.* **11**, 6102 (2020).
12. Liang, Y. Z.*, et al.* Polyamide nanofiltration membrane with highly uniform sub-nanometre pores for sub-1 angstrom precision separation. *Nat. Commun.* **11**, 2015 (2020).
13. Sarkar, P., Modak S. & Karan S. Ultraselective and highly permeable polyamide nanofilms for ionic and molecular nanofiltration. *Adv. Funct. Mater.* **31**, 8 (2021).
14. Zhang, H. Z., Xu Z. L., Ding H. & Tang Y. J. Positively charged capillary nanofiltration membrane with high rejection for Mg^{2+} and Ca^{2+} and good separation for Mg^{2+} and Li^{+}. *Desalination* **420**, 158 (2017).
15. Xu, P.*, et al.* Positive charged PEI-TMC composite nanofiltration membrane for separation of Li^{+} and Mg^{2+} from brine with high Mg^{2+}/Li^{+} ratio. *Desalination* **449**, 57-68 (2019).
16. Li, W., Shi C., Zhou A., He X., Sun Y. & Zhang J. A positively charged composite nanofiltration membrane modified by EDTA for $LiCl/MgCl_2$ separation. *Sep. Purif. Technol.* **186**, 233 (2017).
17. Guo, C.*, et al.* Ultra-thin double Janus nanofiltration membrane for separation of Li^{+} and Mg^{2+}: “Drag” effect from carboxyl-containing negative interlayer. *Sep. Purf. Technol.* **230**, 115567-115581 (2020).
18. Li, X., Zhang C., Zhang S., Li J., He B. & Cui Z. Preparation and characterization of positively charged polyamide composite nanofiltration hollow fiber membrane for lithium and magnesium separation. *Desalination* **369**, 26-36 (2015).
19. Shen, Q., Xu S. J., Xu Z. L., Zhang H. Z. & Dong Z. Q. Novel thin-film nanocomposite membrane with water-soluble polyhydroxylated fullerene for the separation of Mg^{2+}/Li^{+} aqueous solution. *J. Appl. Polym. Sci.* **136**, 48029 (2019).
20. Wu, H. H.*, et al.* A novel nanofiltration membrane with [MimAP][Tf_2N] ionic liquid for utilization of lithium from brines with high Mg^{2+}/Li^{+} ratio. *J. Membr. Sci.* **603**, 117997 (2020).
21. Xu, P., Hong J., Qian X. M., Xu Z. Z., Xia H. & Ni Q. Q. “Bridge” graphene oxide modified positive charged nanofiltration thin membrane with high efficiency for Mg^{2+}/Li^{+} separation. *Desalination* **488**, 114522 (2020).
22. Yang, Z., Fang W. X., Wang Z. Y., Zhang R. L., Zhu Y. Z. & Jin J. Dual-skin layer nanofiltration membranes for highly selective Li^{+}/Mg^{2+} separation. *J. Membr. Sci.* **620**, 118862 (2021).
23. Bi, Q., Zhang C., Liu J., Liu X. & Xu S. Positively charged zwitterion-carbon nitride functionalized nanofiltration membranes with excellent separation performance of Mg^{2+}/Li^{+} and good antifouling properties. *Sep. Purf. Technol.* **257**, 117959 (2021).
24. Xu, P., Hong J., Xu Z. Z., Xia H. & Ni Q. Q. Novel aminated graphene quantum dots ($GQDs\text{-}NH_2$)-engineered nanofiltration membrane with high Mg^{2+}/Li^{+} separation efficiency. *Sep. Purf. Technol.* **258**, 118042 (2021).
25. Guo, C. S.*, et al.* Amino-rich carbon quantum dots ultrathin nanofiltration membranes by double “one-step” methods: Breaking through trade-off among separation, permeation and stability. *Chem. Eng. J.* **404**, 127144 (2021).

26. Wang, L., *et al.* Novel positively charged metal-coordinated nanofiltration membrane for lithium recovery. *ACS Appl. Mater. Interfaces* **13**, 16906-16915 (2021).
27. Feng, Y., Peng H. & Zhao Q. Fabrication of high performance Mg^{2+}/Li^{+} nanofiltration membranes by surface grafting of quaternized bipyridine. *Sep. Purf. Technol.* **280**, 119848 (2022).
28. Luo, H., Peng H. W. & Zhao Q. High flux Mg^{2+}/Li^{+} nanofiltration membranes prepared by surface modification of polyethylenimine thin film composite membranes. *Appl. Surf. Sci.* **579**, 152161 (2022).
29. Li, H. W., *et al.* Nanofiltration membrane with crown ether as exclusive Li^{+} transport channels achieving efficient extraction of lithium from salt lake brine. *Chem. Eng. J.* **438**, 135658 (2022).
30. Ni, Y. X., Peng H. W. & Zhao Q. Ultrathin poly(ionic liquid) nanomembranes for high performance Mg^{2+}/Li^{+} separation. *Adv. Mater. Interfaces* **9**, 2201797 (2022).
31. Setiawan, O., Huang Y. H., Abdi Z. G., Hung W. S. & Chung T. S. pH-tunable and pH-responsive polybenzimidazole (PBI) nanofiltration membranes for Li^{+}/Mg^{2+} separation. *J. Membr. Sci.* **668**, 121269 (2023).
32. Ni, H. X., Wang N. X., Yang Y. Y., Shen M. X. & An Q. F. Positively-charged nanofiltration membrane constructed by polyethyleneimine/layered double hydroxide for Mg^{2+}/Li^{+} separation. *Desalination* **548**, 116256 (2023).
33. Li, Y. H., *et al.* Polyamide nanofiltration membranes with rigid–flexible microstructures for high-efficiency Mg^{2+}/Li^{+} separation. *Sep. Purf. Technol.* **306**, 122552 (2023).
34. Xu, P., Hong J., Xu Z. Z., Xia H. & Ni Q. Q. MWCNTs-COOK-assisted high positively charged composite membrane: Accelerating Li^{+} enrichment and Mg^{2+} removal. *Compos. Pt. B* **212**, 108686 (2021).
35. Xu, Y., Peng H. W., Luo H., Zhang Q., Liu Z. T. & Zhao Q. High performance Mg^{2+}/Li^{+} separation membranes modified by a bis-quaternary ammonium salt. *Desalination* **526**, 115519 (2022).
36. Bachman, J. E., Curtiss L. A. & Assary R. S. Investigation of the redox chemistry of anthraquinone derivatives using density functional theory. *J. Phys. Chem. A* **118**, 8852-8860 (2014).
37. Phillips, K. L., Sandler S. I. & Chiu P. C. A method to calculate the one-electronreduction potentials for nitroaromatic compounds based on gas-phase quantum Mechanics. *J. Comput. Chem.* **32**, 226-239 (2011).
38. Marenich, A. V., Cramer C. J. & Truhlar D. G. Universal solvation model based on solute electron density and on a continuum model of the solvent defined by the bulk dielectric constant and atomic surface tensions. *J. Phys. Chem. B* **113**, 6378 (2009).
