# Supplementary material for: Quaternization-spiro design of chlorine-resistant and high-permeance lithium separation membranes
Source: Nat Commun. 2023 Sep 7;14:5483. doi: 10.1038/s41467-023-41169-x (PMC10482931; doi:10.1038/s41467-023-41169-x)
Supplement: Supplementary file 3 — Reporting Summary [file 41467_2023_41169_MOESM3_ESM.pdf]

nature portfolio

Corresponding author(s): Qiang Zhao

Last updated by author(s): Aug 19, 2023

# Reporting Summary

Nature Portfolio wishes to improve the reproducibility of the work that we publish. This form provides structure for consistency and transparency in reporting. For further information on Nature Portfolio policies, see our Editorial Policies and the Editorial Policy Checklist.

## Statistics

For all statistical analyses, confirm that the following items are present in the figure legend, table legend, main text, or Methods section.

| n/a | Confirmed |
|---|---|
| ☐ | ☒ The exact sample size (*n*) for each experimental group/condition, given as a discrete number and unit of measurement |
| ☐ | ☒ A statement on whether measurements were taken from distinct samples or whether the same sample was measured repeatedly |
| ☐ | ☒ The statistical test(s) used AND whether they are one- or two-sided<br>*Only common tests should be described solely by name; describe more complex techniques in the Methods section.* |
| ☐ | ☒ A description of all covariates tested |
| ☐ | ☒ A description of any assumptions or corrections, such as tests of normality and adjustment for multiple comparisons |
| ☐ | ☒ A full description of the statistical parameters including central tendency (e.g. means) or other basic estimates (e.g. regression coefficient) AND variation (e.g. standard deviation) or associated estimates of uncertainty (e.g. confidence intervals) |
| ☐ | ☒ For null hypothesis testing, the test statistic (e.g. *F*, *t*, *r*) with confidence intervals, effect sizes, degrees of freedom and *P* value noted<br>*Give P values as exact values whenever suitable.* |
| ☐ | ☒ For Bayesian analysis, information on the choice of priors and Markov chain Monte Carlo settings |
| ☐ | ☒ For hierarchical and complex designs, identification of the appropriate level for tests and full reporting of outcomes |
| ☐ | ☒ Estimates of effect sizes (e.g. Cohen's *d*, Pearson's *r*), indicating how they were calculated |

*Our web collection on statistics for biologists contains articles on many of the points above.*

## Software and code

Policy information about availability of computer code

| | |
|---|---|
| Data collection | Gromacs software package was used to conduct the molecule dynamic simulations (Fig. 2d, e, Fig. 4, Supplementary Fig. 6). Gaussian 09 software package was used to evaluate geometries and energetics of molecules (Fig. 6a-d). |
| Data analysis | Zeo++ software was used to analyse cavity size distributions of QSPIP-TMC and PIP-TMC network (Supplementary Fig.9). |

For manuscripts utilizing custom algorithms or software that are central to the research but not yet described in published literature, software must be made available to editors and reviewers. We strongly encourage code deposition in a community repository (e.g. GitHub). See the Nature Portfolio guidelines for submitting code & software for further information.

## Data

Policy information about availability of data

All manuscripts must include a data availability statement. This statement should provide the following information, where applicable:

- Accession codes, unique identifiers, or web links for publicly available datasets
- A description of any restrictions on data availability
- For clinical datasets or third party data, please ensure that the statement adheres to our policy

Data available on request from the corresponding author.

## Research involving human participants, their data, or biological material

Policy information about studies with human participants or human data. See also policy information about sex, gender (identity/presentation), and sexual orientation and race, ethnicity and racism.

| | |
|---|---|
| Reporting on sex and gender | This work doesn't involve human participants, their data or biological material. Thanks! |
| Reporting on race, ethnicity, or other socially relevant groupings | This work doesn't involve human participants, their data or biological material. Thanks! |
| Population characteristics | Please see above. Thanks! |
| Recruitment | This work doesn't involve human participants, their data or biological material. Thanks! |
| Ethics oversight | This work doesn't involve human participants, their data or biological material. Thanks! |

Note that full information on the approval of the study protocol must also be provided in the manuscript.

# Field-specific reporting

Please select the one below that is the best fit for your research. If you are not sure, read the appropriate sections before making your selection.

☐ Life sciences ☐ Behavioural & social sciences ☒ Ecological, evolutionary & environmental sciences

For a reference copy of the document with all sections, see nature.com/documents/nr-reporting-summary-flat.pdf

# Ecological, evolutionary & environmental sciences study design

All studies must disclose on these points even when the disclosure is negative.

| | |
|---|---|
| Study description | We designed a quaternized-spiro piperazine (QSPIP) monomer and translated its beneficial properties into large-area membranes (1 × 2 m2) via interfacial polymerization with trimesoyl chloride. The quaternary ammonium and spiral conformation of QSPIP confer more positive charge and free volume to the membrane, leading to one of the highest permeance (~22 L m-2 h-1 bar-1) compared to state-of-the-art Mg2+/Li+ nanofiltration membranes. Meanwhile, membrane structures are chlorine resistant and membranes performance were reproducible at the membrane module level. |
| Research sample | We prepared all membrane samples by ourselves in lab and we didn't use biological samples. |
| Sampling strategy | The sample of small membrane and large-area membrane was chosen randomly (8 x 8 cm2) for nanofiltration test. Each data (including flux, rejection, roughness, etc) was repeated for at least three times to avoid the accidental error. |
| Data collection | H.W. performed the experimental studies with help from X.F. and J.P. K.C. and X.G. carried out the energy calculation and simulations. |
| Timing and spatial scale | Separation performance was evaluated by cross-flow (rate: 0.5 L min-1) nanofiltration and membranes were stabilized at 6 bar for 30 min before test. Each data was continually collected for at least 1 h. |
| Data exclusions | No data were excluded from the analyses. |
| Reproducibility | X.F. and J.P. succeed to reproduce the experimental findings. |
| Randomization | All membrane samples were chosen randomly for test. |
| Blinding | We did't involve the blinding in this work. All data were collected. |

Did the study involve field work? ☐ Yes ☒ No

# Reporting for specific materials, systems and methods

We require information from authors about some types of materials, experimental systems and methods used in many studies. Here, indicate whether each material, system or method listed is relevant to your study. If you are not sure if a list item applies to your research, read the appropriate section before selecting a response.

## Materials & experimental systems

| n/a | Involved in the study |
|---|---|
| ☒ | ☐ Antibodies |
| ☒ | ☐ Eukaryotic cell lines |
| ☒ | ☐ Palaeontology and archaeology |
| ☒ | ☐ Animals and other organisms |
| ☒ | ☐ Clinical data |
| ☒ | ☐ Dual use research of concern |
| ☒ | ☐ Plants |

## Methods

| n/a | Involved in the study |
|---|---|
| ☒ | ☐ ChIP-seq |
| ☒ | ☐ Flow cytometry |
| ☒ | ☐ MRI-based neuroimaging |
